# The Role of CA125 and HE4 in Uterine Sarcomas: Beyond Diagnosis and Prognosis—A Systematic Review and Case Series from a Single Institution

**DOI:** 10.3390/cancers17091473

**Published:** 2025-04-27

**Authors:** Gianna Barbara Cundari, Laura Feole, Corrado Terranova, Carlo De Cicco Nardone, Roberto Montera, Daniela Luvero, Federica Guzzo, Arianna Martinelli, Violante Di Donato, Roberto Angioli, Francesco Plotti

**Affiliations:** 1 Research Unit of Gynaecology, Department of Medicine and Surgery, Università Campus Bio-Medico Di Roma, Via Alvaro del Portillo 21, 00128 Rome, Italy; l.feole@unicampus.it (L.F.); f.plotti@policlinicocampus.it (F.P.); 2 Department of Gynaecology, Fondazione Policlinico Universitario Campus Bio-Medico, Via Alvaro del Portillo 200, 00128 Rome, Italy; 3 Department of Maternal and Child Health and Urological Sciences, Sapienza University of Rome, Policlinico Umberto I, 00185 Rome, Italy

**Keywords:** uterine sarcoma, gynecological cancer, serum biomarkers, CA125, HE4

## Abstract

***Background***: Serum biomarkers such as Carcinoma Antigen 125 (CA125) and Human Epididymis Protein 4 (HE4) are widely used in the diagnosis and prognosis of gynecological malignancies. Serum biomarkers such as CA125 and HE4 represent essential tools in improving early detection, risk stratification, and therapeutic decision-making for gynecological malignancies. However, their role in identifying uterine sarcomas remains debated. This systematic review and case series aims to examine the diagnostic and prognostic significance of CA125 and HE4 in uterine sarcomas. ***Methods***: A systematic review was performed on studies investigating serum CA125 and HE4 levels in uterine sarcomas. A case series of all uterine sarcomas treated at the Campus Bio-Medico Gynecology Unit of Rome from 2010 to 2020 was analyzed. ***Results***: The analysis of the 11 selected studies allowed us to investigate the role of CA125 in uterine sarcomas. No studies analyzing the role of HE4 in monitoring the disease were found. A total of 16 patients with confirmed uterine leiomyosarcoma were included in our case series. ***Conclusions***: Neither CA125 nor HE4 can currently be considered definitive biomarkers for the diagnosis of uterine leiomyosarcomas. However, they may serve as useful adjuncts in the differential diagnosis between leiomyomas and leiomyosarcomas, particularly in reproductive-age patients.

## Simple Summary

Uterine sarcomas are rare and aggressive tumors with an unfavorable prognosis, making the identification of parameters that could aid in diagnosis and/or prognosis essential. Our study aims to assess the role of serum levels of two biomarkers already used in gynecologic oncology but never previously applied to the diagnosis and prognosis of uterine sarcomas. Our study represents a starting point for further research with larger case series on this topic, which could ultimately lead to earlier diagnosis of these neoplasms and contribute to a better prognostic assessment of affected patients.

## 1. Introduction

Uterine sarcomas are a rare and aggressive group of mesenchymal tumors, accounting for less than 10% of all uterine neoplasms [1]. In Italy, the annual incidence of uterine sarcomas is estimated to be approximately 0.19 cases per 100,000 women, with sarcomas representing only 0.2% of all malignant uterine neoplasms [1]. The prognosis for patients diagnosed with uterine sarcoma is generally poor, with a 5-year survival rate of 51% for stage I, which decreases to 3% for stage IV [1]. Despite the increasing global interest, which has led to the development of new guidelines for the management of these patients, their heterogeneity and the lack of reliable preoperative diagnostic tools continue to present significant challenges in clinical practice [2].

One of the most urgent clinical needs in this field is the ability to accurately distinguish benign uterine leiomyomas from malignant uterine leiomyosarcomas in the preoperative phase [3,4]. This distinction is crucial, as the surgical management of these two entities is very different. Leiomyomas can be treated with minimally invasive procedures, sometimes involving power morcellation, whereas uterine sarcomas require en-bloc radical surgery to prevent abdominal tumor dissemination [5]. Power morcellation is, in fact, a well-documented risk for disease spread [6]. The absence of a reliable predictive system often leads to overtreatment of benign conditions and inadvertent morcellation of malignant tumors, worsening patient prognosis.

The identification of a biomarker or predictive model that effectively differentiates these tumors in the preoperative phase would have significant clinical implications, optimizing surgical decisions and improving patient outcomes. Among the serum biomarkers explored in gynecologic oncology, Cancer Antigen 125 (CA125) and Human Epididymis Protein 4 (HE4) have been extensively studied in epithelial ovarian cancer and endometrial cancer [7,8]. However, their diagnostic and prognostic role in uterine sarcomas remains controversial. CA125, a glycoprotein associated with peritoneal irritation, is primarily used to monitor treatment response, and detect recurrence in patients with ovarian and/or peritoneal carcinoma [9]. However, its specificity is limited. CA125 has been evaluated in various studies for its potential role in the diagnosis, prognosis, and monitoring of uterine sarcomas. On the other hand, HE4 has emerged as a promising biomarker in gynecologic oncology, particularly in ovarian and endometrial cancers [7,8,10]. HE4 is a protein involved in sperm maturation but has also been identified as a tumor marker in ovarian and/or peritoneal carcinoma and endometrial carcinoma [7,8,10]. However, its role in uterine sarcomas remains largely unexplored, with no data available regarding its diagnostic and prognostic utility.

The limitations in scientific research and clinical application of these biomarkers are mainly related to the variability of results across different studies, the low specificity and sensitivity of CA125, the scarcity of data regarding HE4 in gynecologic tumors, and the complete absence of data on the use of HE4 in uterine sarcomas. A possible solution to improve diagnostic reliability could be a multimodal approach, integrating CA125 and HE4 with other diagnostic techniques, such as radiological imaging (e.g., magnetic resonance imaging) or genomic analysis, in order to reduce uncertainties and improve predictivity.

This study aims to provide a systematic and comprehensive review of the literature regarding the role of CA125 and HE4 in uterine sarcomas, evaluating their potential diagnostic and prognostic roles. Additionally, we present a case series from our institution, analyzing serum levels and the clinical implications of CA125 and HE4 in a cohort of patients with histologically confirmed uterine sarcoma. By integrating existing evidence with our real-world data, we aim to clarify the clinical utility of these biomarkers and their potential role in guiding therapeutic decisions.

## 2. Materials and Methods

### 2.1. Systematic Literature Review

This systematic review was conducted according to PRISMA (Preferred Reporting Items for Systematic Reviews and Meta-Analyses) guidelines. The systematic review registration ID is from PROSPERO: 1038149. A literature search was conducted using the PubMed database, with the keywords “uterine sarcoma, CA125” and “uterine sarcoma, HE4” with no limitations on publication date. The database was screened for search terms appearing in either titles or abstracts. The outcome of our systematic review was to evaluate the role of the biomarkers CA125 and HE4 in uterine sarcomas, including their value in diagnosis, prognosis, and disease monitoring.

The identified articles were initially screened based on the following inclusion criteria:

- Original studies (prospective, retrospective, observational, or case series).
- Studies analyzing the role of CA125 in uterine sarcomas (diagnosis, prognosis, monitoring).
- Studies published in English.

Then, we excluded the following:

- Studies based only on carcinosarcomas, due to their partially epithelial origin.
- Studies on extrauterine sarcomas.
- Studies not reporting data on CA125/HE4 and uterine sarcomas.
- Reviews, letters to the editor, and abstracts without complete data.

Study selection was independently performed by three authors (G.B.C., L.F., and F.P.). Titles and abstracts were screened for eligibility based on the inclusion and exclusion criteria, and any titles or abstracts deemed ineligible were excluded. If a study was deemed relevant, the manuscript was obtained and reviewed in full text. Data were collected using an Excel spread sheet. All authors independently assessed the quality of the studies, taking into consideration representativeness and quality of data. Two reviewers (C.T. and A.M.) independently assessed the risk of bias. In case of disagreement, a third reviewer (R.A.) was consulted.

For each included study, the following data were collected:

- Author and year of publication.
- Study type (retrospective, prospective, case report, etc.).
- Number of patients included.
- Role of CA125 or HE4 (diagnosis, prognosis, disease monitoring).
- Main findings.

**Figure 1:**
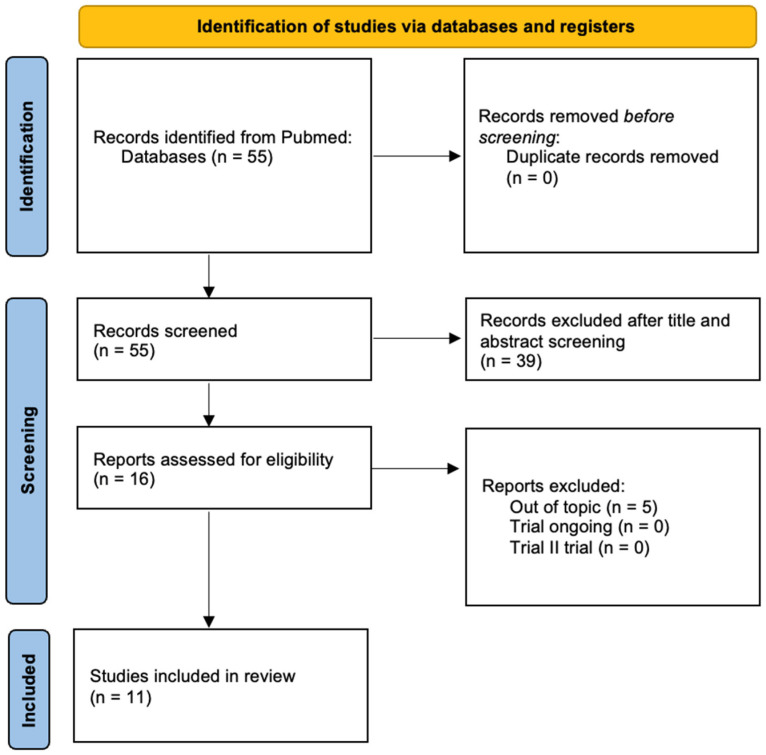
Flowchart for study selection of the review.

The data are summarized in a review table and analyzed qualitatively as Figure 1.

Due to heterogeneity in study populations and methodologies, no quantitative meta-analysis was conducted.

#### Risk of Bias

The risk of bias was assessed using the Newcastle–Ottawa scale (NOS) for cross-sectional studies [11]. No risk of bias was assessed for case reports. The results from the NOS were converted into the Agency for Healthcare Research and Quality (AHRQ) standards of “good”, “fair”, and “poor” quality.

Thresholds for converting the Newcastle–Ottawa scales to AHRQ standards (good, fair, and poor):

- Good quality: 3 or 4 stars in the selection domain AND 1 or 2 stars in the comparability domain AND 2 or 3 stars in the outcome/exposure domain.
- Fair quality: 2 stars in the selection domain AND 1 or 2 stars in the comparability domain AND 2 or 3 stars in the outcome/exposure domain.
- Poor quality: 0 or 1 star in the selection domain OR 0 stars in the comparability domain OR 0 or 1 stars in the outcome/exposure domain.

### 2.2. Case Series

A retrospective study was conducted on a case series of patients diagnosed with uterine leiomyosarcoma who underwent surgery at the Gynecology Unit of Campus Bio-Medico University of Rome. Clinical and histopathological data were collected and analyzed to evaluate patients’ demographic, clinical, and laboratory characteristics.

Patients included were those with a confirmed histological diagnosis of uterine leiomyosarcoma, including the following subtypes: conventional leiomyosarcoma, low-grade and high-grade leiomyosarcoma, undifferentiated leiomyosarcoma, myxoid leiomyosarcoma, and spindle cell leiomyosarcoma.

Patients were selected by reviewing surgical and histopathological hospital records. All patients were aged 18 to 90 years and had undergone myomectomy and/or hysterectomy at our institution between 2010 and 2025. They underwent follow-up gynecological visits every six months for the first ten years, then annually. Exclusion criteria included uncertain histological diagnosis, loss to follow-up, incomplete data, synchronous neoplasms, pregnancy, endometriosis, pelvic inflammatory disease (PID), chronic renal failure, inflammatory bowel disease, and chronic liver disease.

All collected information was entered into a dedicated database. The recorded data included age at diagnosis, menopausal status, parity, family history of gynecological malignancies, body mass index (BMI), histological subtype of sarcoma, preoperative serum CA125 levels (U/mL), preoperative serum HE4 levels (pmol/L), overall survival (OS), and progression-free survival (PFS). OS was defined as the duration between diagnosis or treatment initiation and death from any cause, while PFS was defined as the time from diagnosis or treatment initiation until disease progression or death from any cause. Preoperative serum tumor marker levels were obtained from blood tests. Histopathological analysis of surgical specimens was performed by expert gynecologic oncopathologists following the WHO classification criteria for uterine sarcomas [12]. We also attempted to apply the “ROMA score”, which is described in the literature for assessing the risk of epithelial ovarian cancer in patients with a pelvic mass. Until now, the ROMA score has never been applied to suspected uterine neoplasms for sarcoma [13,14].

After review by the local ethics committee, our study was classified as exempt from Internal Review Board oversight since it was a non-interventional observational study (without randomization), reflecting its retrospective design. The study was conducted in compliance with Good Clinical Practice and the Declaration of Helsinki. According to our internal protocols, all patients provided informed consent for the use of their clinical data for research purposes at hospital admission. Thus, all patients signed a written consent form in accordance with Italian Privacy Law (675/96). Ethical considerations ensured patient privacy and confidentiality throughout the study.

#### Statistical Analysis

The data were analyzed using descriptive statistical methods, reporting means, medians, and ranges for continuous variables and absolute and percentage frequencies for categorical variables. The analyses were performed using dedicated statistical software (Python, version 3.13.2). To evaluate overall survival (OS) and progression-free survival (PFS), Kaplan–Meier curves were constructed, and comparisons between groups were made using the log-rank test. In addition to survival tests, further comparisons were conducted. For continuous variables (e.g., age, quantitative biomarkers), statistical tests such as t-tests or Mann–Whitney tests were applied, depending on data distribution. For categorical variables (such as histological subtype and menopausal status), contingency analyses (e.g., chi-square test) were performed to assess potential associations. A *p*-value <0.05 was considered statistically significant.

## 3. Results

### 3.1. Systematic Literature Review

From our search, 55 articles that referred to CA125 were identified. The search for HE4 did not produce any relevant results. After title/abstract screening, 16 eligible articles were assessed for eligibility. According to inclusion and exclusion criteria, 5 articles were excluded because they were out of topic. At the end of the screening, a total of 11 studies were included in our review as Table 1.

The results of the assessment of risk of bias for cross-sectional studies are presented in Table 2.

The analysis of the 11 selected studies allowed us to investigate the role of CA125 in uterine sarcomas in terms of differential diagnosis, prognosis, and disease monitoring. Eight studies (72%) analyzed the role of tumor marker CA125 in the diagnosis of leiomyosarcomas, while three studies (18%) analyzed its prognostic role. No studies analysing the role of HE4 in monitoring the disease were found.

One of the most studied aspects in the literature is the role of CA125 in the preoperative differential diagnosis of leiomyomas and leiomyosarcomas, which is crucial for determining the type and method of surgical intervention (laparoscopy vs. laparotomy). A retrospective study highlighted that preoperative CA125 levels were significantly higher in patients with leiomyosarcoma than in those with leiomyoma [15]. Additionally, a case report published in 2024 showed that CA125 levels can be markedly elevated in patients with massive uterine sarcomas [16]. However, the authors demonstrated that CA125 does not significantly change in patients with early-stage leiomyosarcoma, failing to provide an adequate differential diagnosis between early-stage leiomyosarcoma and benign leiomyoma. This reduces CA125’s reliability as a standalone preoperative diagnostic tool.

Confirming the limitations of this biomarker, another study conducted in 2009 on a large cohort of 2382 patients with leiomyoma and 26 with uterine sarcoma concluded that CA125 has no predictive value in differential diagnosis [17]. Furthermore, no clear correlation emerged between disease stage and CA125 levels, making its preoperative use unreliable even in this context. A more recent study, published in 2010 by Kim et al., compared CA125 and the neutrophil-to-lymphocyte ratio (NLR) in the preoperative differential diagnosis of uterine leiomyosarcomas and leiomyomas [18]. The authors concluded that NLR has higher sensitivity and specificity in distinguishing uterine sarcomas from leiomyomas, suggesting that alternative biomarkers may provide more useful information than CA125 alone.

Several studies have explored the possible prognostic value of CA125, attempting to determine whether elevated levels correlate with reduced survival or increased disease aggressiveness. A study published by Yang et al. in 2024 found that, among the various factors analyzed, OS in patients with uterine sarcoma was significantly affected only by CA125 levels and tumor recurrence [19]. In the same analysis, PFS was associated only with recurrence, highlighting the potential role of CA125 as a prognostic biomarker. Similarly, a retrospective study conducted on 29 patients with uterine sarcoma and 34 with carcinosarcoma confirmed an association between elevated CA125 levels and poor prognosis [20]. Besides CA125, other significant prognostic factors included tumor size, lymph node positivity, high mitotic activity, vascular invasion, and high BMI, suggesting that the biomarker could be part of a multifactorial prognostic panel. Additional data seem to support this association: Zhang, in a study published in 2019, showed that the risk of death increases proportionally with minimum and median CA125 levels, indicating that even small variations in this biomarker’s levels can reflect disease progression [21].

Although CA125 is traditionally considered a serum biomarker, some studies have evaluated its expression in tumor tissue through immunohistochemical analyses. One study examined 17 uterine leiomyosarcoma samples and found that CA125 was not expressed in any of them [22]. The authors hypothesized that the increased serum biomarker levels in some patients do not originate directly from neoplastic cells but rather from secondary activation of mesothelial cells. These findings were confirmed by another study conducted by Duk et al., which showed that CA125 is not expressed in uterine sarcoma tissues, unlike in the epithelial component of Müllerian mixed tumors, reinforcing the hypothesis that the biomarker is not directly produced by uterine leiomyosarcoma cells [23].

The use of CA125 as a monitoring marker in uterine sarcomas has been less studied, but some evidence suggests that it may help detect recurrences. A case report described that CA125 levels were elevated at diagnosis, normalized after chemotherapy, and rose again at recurrence, suggesting that the biomarker may reflect disease progression [24]. However, a larger study found that although CA125 was elevated in 75% of patients with stage I uterine sarcomas with extrauterine spread, its monitoring during chemotherapy was unreliable in assessing treatment response [25]. This finding suggests that while CA125 may help suspect a recurrence, it is not an accurate indicator of treatment response.

### 3.2. Case Series

#### 3.2.1. Clinical and Histopathological Characteristics

A total of 20 patients who underwent myomectomy and/or hysterectomy at the Gynecology Unit of Campus Bio-Medico University of Rome between 2010 and 2025 with a histological diagnosis of uterine leiomyosarcoma were recruited. One patient was excluded due to a synchronous tumor (uterine leiomyosarcoma and high-grade serous ovarian carcinoma), two patients were lost to follow-up, and one was excluded due to chronic renal failure. A total of 16 patients with confirmed uterine leiomyosarcoma were included in the study, as shown in Table 3.

The mean age at diagnosis was 57 years (range: 46–83). The average BMI was 27.1 kg/m^2^ (range: 20–42). The median parity was 1 (range: 0–3), and only one patient (6.3%) had a family history of gynecological malignancies. The most common histological subtype was conventional uterine leiomyosarcoma (*n* = 6, 37.5%), followed by spindle cell leiomyosarcoma (*n* = 4, 25%), low-grade leiomyosarcoma (*n* = 1, 6.3%), myxoid leiomyosarcoma (*n* = 2, 12.5%), and high-grade leiomyosarcoma (*n* = 1, 6.3%).

#### 3.2.2. Diagnosis: Role of CA125 and HE4

We considered the following cut-off values for the analyzed tumor markers, above which they were classified as positive: 35 U/mL for CA125, 70 pmol/L for HE4 in premenopausal women, and 140 pmol/L for HE4 in postmenopausal women [26,27]. Nine out of sixteen patients (56%) had elevated CA125 levels. Among them, all premenopausal patients (100%) and only 3 out of 10 postmenopausal patients (30%) had positive CA125. Seven out of sixteen patients (43%) had elevated HE4 levels. Among them, 5 out of 6 premenopausal patients (83%) but only 2 out of 10 postmenopausal patients (20%) had positive HE4. Only 5 out of 16 patients (31%) showed an alteration in the combination of CA125 and HE4; notably, all of them were premenopausal. Specifically, among premenopausal patients, 5 out of 6 (83%) exhibited an alteration in the combined CA125 and HE4 levels.

Regarding the calculation of the ROMA score in our case series, we consider values ≥ 12.5% in premenopausal women and ≥14.5% in postmenopausal women as predictive of a high risk of malignant neoplasm. In 11 out of 16 patients (68%), we found a score predictive of a high risk of malignant neoplasm. Among these, 5 out of 6 premenopausal patients (83%) and 6 out of 10 postmenopausal patients (60%) had a positive ROMA score.

#### 3.2.3. Prognosis: Overall Survival (OS) and Progression-Free Survival (PFS)

The median overall survival (OS) was 57 months (range: 9–171 months), while the median PFS was 13 months (range: 9–94 months). At the end of the follow-up period, 10 patients (62.5%) were still alive, while 6 patients (37.5%) had died.

The analysis of different histological subtypes revealed variability in OS and PFS:

- Spindle cell leiomyosarcoma had the shortest PFS, with a median of 13 months (range: 9–94 months).
- The low-grade subtype exhibited a longer PFS (median: 12 months), while the high-grade subtype had a PFS of 45 months.
- Spindle cell leiomyosarcoma showed a median OS of 171 months for a censored case (patient still alive) and 13 months for a non-censored case (deceased patient).
- Overall survival was highest for the spindle cell leiomyosarcoma subtype, with one censored case at 171 months.

**Figure 2:**
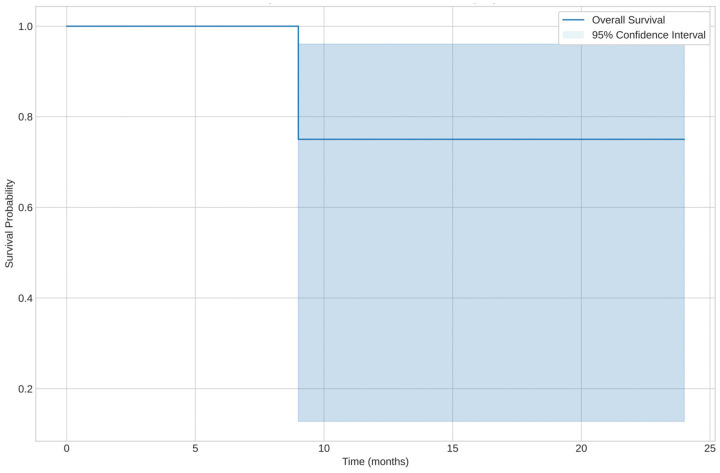
Kaplan–Meier curve for Overall Survival (OS).

The median estimated OS was approximately 24 months. Kaplan–Meier analysis showed different survival curves when stratifying by histological subtype, as shown in Figure 2.

**Figure 3:**
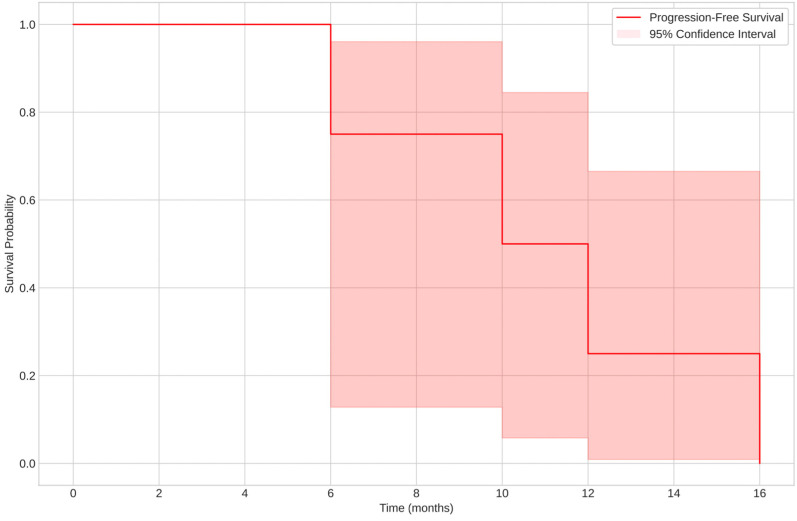
Kaplan–Meier curve for Progression-Free Survival (PFS).

The estimated median PFS was around 16 months, with significant differences between groups based on menopausal status and other clinical variables, as shown in Figure 3.

The log-rank test indicated a statistically significant difference between some groups, suggesting that histological subtype (*p* ≈ 0.04) and age at diagnosis (*p* ≈ 0.03) were associated with survival differences as Table 4.

No statistically significant prognostic associations were found between CA124 and HE4 and leiomyosarcomas. It was found that certain clinical characteristics of the patients, including age at diagnosis and histological subtype, play a significant role in disease progression, influencing both OS and PFS.

## 4. Discussion

Uterine leiomyosarcomas represent a rare yet aggressive gynecologic malignancy, for which early diagnosis and accurate prognostic stratification are crucial. In our literature review, we analyzed the role of CA125 and HE4 as potential serum biomarkers for the diagnosis and prognosis of leiomyosarcomas, highlighting conflicting data and a still limited use in clinical practice.

From the available data, CA125, although widely used in the management of ovarian tumors [26], shows low diagnostic specificity in leiomyosarcomas, with elevated levels observed in various benign conditions or other gynecologic malignancies [15,17]. Some studies suggest that preoperative CA125 elevation may aid in distinguishing leiomyomas from leiomyosarcomas [15], while others argue that its diagnostic utility is limited due to significant overlap between benign and malignant conditions [16,17]. Similarly, prognostic studies have reported conflicting findings regarding CA125 as an independent predictor of survival, with some evidence indicating an association between high serum levels and advanced disease, poor prognosis, or tumor recurrence [19,20,21]. Moreover, immunohistochemical analyses have shown that, although serum CA125 may be elevated in some uterine sarcomas, its expression in tumor tissue is often absent, raising questions about the origin of these increased levels [22,23]. Some studies suggest an association between elevated CA125 and more aggressive leiomyosarcoma behavior [19], but the isolated use of this biomarker appears insufficient for an accurate diagnosis.

On the other hand, HE4 has been proposed as a more specific biomarker for malignant gynecologic neoplasms, particularly serous ovarian carcinoma [7,8,28,29,30]. However, data on its diagnostic and prognostic role in leiomyosarcomas are limited.

In our case series, we observed an expression profile of CA125 and HE4 that partially reflects the findings reported in the literature. Specifically, CA125 levels were elevated in some patients with high-grade leiomyosarcoma, suggesting a possible correlation with the tumor’s aggressive behavior. However, interindividual variability and overlap with other conditions limit the use of CA125 as a standalone diagnostic marker.

Regarding HE4, our data suggest an expression trend that may have prognostic significance. However, even in this case, the sensitivity and specificity of the test remain open issues, requiring further investigation in larger cohorts to validate its clinical application. Considering the limitation given by the small simple size, our findings indicate a higher sensitivity of both tumor markers (CA125 and HE4), particularly in premenopausal patients. Specifically, we observed a diagnostic sensitivity of CA125 in premenopausal women of 100% and an HE4 sensitivity of 83% in the same patient group. Conversely, the combination of both markers showed a diagnostic sensitivity of 83% in premenopausal women. Subgroup analysis revealed that the ROMA score had higher sensitivity in premenopausal patients (83%) compared to postmenopausal patients (60%). These data suggest that the ROMA score, CA125, and HE4 may have greater diagnostic accuracy in reproductive-age women, while further refinement of interpretation criteria may be needed for postmenopausal patients.

### 4.1. Strengths and Limitations

As the principal strength of our work, this is the first study that analyzes the role of CA125, HE4, and ROMA score in the diagnosis and prognosis of uterine sarcomas. Our study combines a systematic review of the literature with a retrospective case series, providing both a comprehensive overview and a concrete clinical analysis on the topic. The subject addressed may have practical implications in clinical settings, particularly by improving preoperative diagnosis and guiding surgical decision-making in cases of suspected leiomyoma versus leiomyosarcoma.

Our data should be interpreted with caution due to certain study limitations, including the relatively small sample size and the lack of long-term follow-up to confirm the predictive reliability of the analyzed variables. Moreover, the variability of tumor marker levels in different benign and malignant gynecologic conditions represents an additional potential confounding factor. Additionally, the literature on this argument is scarce: only 11 studies can be included in the analysis. None of the studies included in the literature review investigated HE4 in the context of uterine sarcomas, and the data derived from the case series remain exploratory. The absence of a meta-analysis, due to heterogeneity among studies, limits the overall conclusiveness of the systematic review.

### 4.2. Future Perspectives

The prognostic role of CA125 and HE4 warrants further investigation, especially in contexts where a more accurate risk stratification could guide therapeutic decisions. Extensive prospective studies are required to more clearly establish the clinical value of these biomarkers and their potential role in the management of leiomyosarcomas. It would be useful to develop an ideal score, which includes CA125 and HE4 among the variables, that can help in preoperative diagnostic guidance and can best judge therapeutic choices.

## 5. Conclusions

This is the first study in literature that analyzes the role of CA125, HE4, and ROMA score in the diagnosis and prognosis of uterine sarcomas. From our literature analysis and preliminary data, neither CA125 nor HE4 can currently be considered definitive biomarkers for the diagnosis of uterine leiomyosarcomas. However, they may serve as useful adjuncts in the differential diagnosis between leiomyomas and leiomyosarcomas, particularly in reproductive-age patients. Large-scale prospective studies are necessary to better define the clinical utility of these biomarkers and their potential impact on leiomyosarcoma management.

## Figures and Tables

**Table 1:** Studies’ characteristics.

Author, Year	Journal	Country	Study Design	Number of Pts ^1^	Role of CA125	Main Findings
Juang C.M. et al., 2006 [15]	Eur J Gynaecol Oncol	Taiwan	Comparative study	42	Diagnosis	Preoperative serum CA125 had a potential role in the differential diagnosis between early-stage and advanced-stage uterine leiomyosarcoma.
Das S. et al., 2024 [16]	Pan Afr Med	India	Case report	1	Diagnosis	CA125 level and LDH level were elevated.
Yilmaz N. et al., 2009 [17]	Eur J Gynaecol Oncol	Turkey	Retrospective study	26	Diagnosis	In the differential diagnosis of myoma and uterine sarcoma, the preoperative serum CA125 level did not have any predictivity. There was no association between staging and CA125 in uterine sarcomas.
Kim H.S. et al., 2010 [18]	Eur J Surg Oncol	Republic of Korea	Observational study	20	Diagnosis	There was no difference in the values of serum CA125 levels between preoperative and recurrence times; preoperative and end-of-the-study times; response evaluation and end-of-the-study times, compared with values of the NLR.
Yang L. et al., 2024 [19]	Technol Cancer Res Treat	China	Comparative study	57	Prognosis	Only CA125 and tumor recurrence remained significant predictors of OS, while only tumor relapse had prognostic significance for PFS (*p* < 0.05)
Visnovsky J. et al., 2015 [20]	Neuro Endocrinol Lett	Slovakia	Retrospective study	29	Prognosis	Highly statistically significant values for the inverse correlation between survival and tumor size, positive lymph nodes, high mitotic activity, vascular invasion, positive peritoneal cytology, elevated CA125, smoking, and BMI in sarcoma (*p* < 0.001 for all factors).
Zhang Y. et al., 2019 [21]	Cancer Manag Res.	China	Retrospective observational study	40	Prognosis	The probability of death increased by 1.234-fold (95% CI: 1.032–1.476) or 1.016-fold (95% CI: 1.004–1.028) for each unit of increase in the minimum or average value of CA125.
Menczer J. et al., 2014 [22]	Isr Med Assoc J	Israel	Retrospective study	17	Diagnosis	CA125 was not immunohistochemically expressed in the tissue of any LMS tumors examined. The origin of elevated serum CA125 in some of these tumors is therefore not in its tissue and remains unknown.
Duk J.M. et al., 1994 [23]	Int J Gynecol Cancer	Netherlands	Observational prospective study	33	Diagnosis	Sarcoma cells were completely negative for CA125, whereas positivity was observed in the epithelial component of mixed Müllerian tumors. The source of the elevated serum CA125 levels in patients with uterine sarcoma may be stimulated mesothelial cells.
Vigon A. et al., 2005 [24]	IJGC	Italy	Case report	1	Diagnosis	The level of serum CA125 was high at diagnosis, within normal limits after the fifth cycle of chemotherapy, and subsequently increased again at recurrence.
Patsner B. et al., 1988 [25]	Cancer	New York state	Observational study	11	Diagnosis	Elevated preoperative serum CA125 levels were found. Serial levels during chemotherapy inconsistently reflected response to treatment and proved to have limited clinical value.

^1^ Pts: patients.

**Table 2:** Risk of bias assessment using Newcastle–Ottawa scale (NOS).

Study	Year	Selection (*/4)	Comparability (*/2)	Exposure (*/3)	Total (*/9)	Estimated Risk of Bias
Juang et al. [15]	2006	***	*	**	6	Good
Yilmaz et al. [17]	2009	***	*	*	5	Fair
Kim et al. [18]	2010	****	**	**	8	Good
Yang et al. [19]	2024	****	**	**	8	Good
Visnovsky et al. [20]	2015	**	*	**	5	Fair
Zhang et al. [21]	2019	***	**	**	7	Good
Menczer et al. [22]	2014	**	*	*	4	Poor
Duk et al. [23]	1994	**	*	*	4	Poor
Patsner et al. [25]	1988	*	-	*	2	Poor

* = score 1 in one item within each section, ** = score 1 in two items within each section, *** = score 1 in three items within each section, **** = score 1 in four items within each section.

**Table 3:** Patients’ characteristics from our case series.

Patient	Age at Diagnosis (years)	Menopause	Parity	Family History for Gyn Cancer	BMI	Histology	CA125 (U/mL)	HE4 (pmol/L)	ROMA SCORE	OS (months)	PFS (months)
1	46	No	0	No	22	Leiomyosarcoma conventional type	76	162	59.38%	8+	-
2	43	No	1	No	21	Low-grade leiomyosarcoma	86	50	8.24%	12+	-
3	56	Yes	1	No	30	Undifferentiated leiomyosarcoma	17	36	9.2%	12+	-
4	44	No	2	No	22	Undifferentiated leiomyosarcoma	86	444	94.2%	24+	-
5	70	Yes	1	No	22	Undifferentiated leiomyosarcoma	37	103	34.83%	30+	-
6	58	Yes	0	Yes	27	Leiomyosarcoma conventional type	68	37	22.34%	17+	-
7	55	Yes	0	No	31	Myxoid leiomyosarcoma	27	115	32.24%	27+	-
8	54	Yes	0	No	37	Spindle cell leiomyosarcoma	31	29	11.16%	47+	-
9	54	Yes	0	No	20	Leiomyosarcoma conventional type	206	122	69.13%	13	13
10	55	Yes	2	Yes	31	Low-grade leiomyosarcoma	15	42	9.79%	98+	-
11	51	Yes	0	No	24	High-grade leiomyosarcoma	20	45	12.58%	57	45
12	80	Yes	2	No	33	Leiomyosarcoma conventional type	27	250	51.62%	8	8
13	43	No	0	No	20	Leiomyosarcoma conventional type	132	179	65.74%	118	94
14	83	Yes	3	No	26	Spindle cell leiomyosarcoma	78	234	68.41%	3+	-
15	51	No	2	No	25	Leiomyosarcoma conventional type	156	187	68.27%	9	9
16	46	No	2	No	42	Leiomyosarcoma conventional type	120	221	75.9%	171+	-

BMI: Body Mass Index; OS: Overall Survival; PFS: Progression-Free Survival. The “+” sign next to the Overall Survival value indicates that the data are censored (the patient is still alive at the last follow-up).

**Table 4:** Statistical analysis: correlation between patients’ characteristics, OS, and PFS.

Clinical Characteristics	OS (*p*-Value)	PFS (*p*-Value)
Age at diagnosis	0.03	0.04
Menopause	0.06	0.08
Parity	0.15	0.2
Family history for gyn cancer	0.5	0.4
BMI	0.07	0.09
Histology	0.04	0.04
CA125	0.1	0.12
HE4	0.12	0.11

BMI: Body Mass Index; OS: Overall Survival; PFS: Progression-Free Survival.

## Data Availability

The original contributions presented in this study are included in the article. Further inquiries can be directed to the corresponding author.
